# Pulmonary haptoglobin (pHp) is part of the surfactant system in the human lung

**DOI:** 10.1186/1746-1596-7-158

**Published:** 2012-11-20

**Authors:** Mahdi Abdullah, Torsten Goldmann

**Affiliations:** 1 Clinical and Experimental Pathology, Research Center Borstel, Airway Research Center North (ARCN), Member of the German Center for Lung Research, Parkallee 3, D-23845, Borstel, Germany

**Keywords:** Haptoglobin, Lung, Surfactant, Protein

## Abstract

**Abstract:**

Since the existence of pHp was demonstrated, it has been shown that this molecule and its receptor CD163 are regulated by different stimuli. Furthermore, a comparably fast secretion of pHp was described as well as the immuno-stimulatory effects. The intention of this study was to elucidate the role of pHp in the human lungs further. Here we show, by means of confocal microscopy and immune-electron-microscopy, a clear co-localization of pHp with Surfactant protein-B in lamellar bodies of Alveolar Epithelial Cells Type II. These results are underlined by immunohistochemical stainings in differently fixed human lung tissues, which show pHp in vesicular and released form. The images of the released form resemble the intended position of surfactant in the human alveolus. pHp is secreted by Alveolar epithelial cells type II as previously shown. Moreover, pHp is co-localized with Surfactant protein-B. We conclude that the presented data shows that pHp is a native part of the surfactant system in the human lung.

**Virtual slides:**

http://www.diagnosticpathology.diagnomx.eu/vs/2563584738239912.

## Background

We recently reported that the plasma protein haptoglobin (Hp) is also synthesized and expressed in the human lung [1]. We described expression of Hp-protein in Lung cancer (mainly in Adenocarcinomas of the lung), Alveolar Epithelial Cells type II (AECII), alveolar macrophages and bronchial epithelia. The synthesis of pulmonary Hp (pHp) was discovered in screening experiments with transcriptome-chips and validated by RT-PCR [2].

Due to the known immuno-modulatory effects of Hp besides of its primary function in the breakdown of hemoglobin, we analyzed the role of pHp further.

Cell specific synthesis of pHp was addressed by RNA-in situ hybridization, which revealed pHp transcription in AECII and alveolar macrophages [1]. A possible secretion of pHp by pulmonary cells has been addressed utilizing human lung explants and A549 cells. We showed that pHp is indeed secreted by lung tissues and A549 cells. This secretion depends on different stimuli like LPS, IL6, Pam3Cys-Ser-(Lys)4, and, interestingly, dexamethasone [1]. Secretion of pHp happens comparably fast, it was measurable in supernatants already after ten minutes of stimulation, which is by far faster than the most other immuno-regulatory systems in the lung e.g. Toll-like receptor mediated signaling. It has been shown that A549 cells release Interleukin-8 and monocyte chemotactic protein-1 upon co-stimulation with Hp and LPS, and monocyte derived macrophages release TNF-α, Interleukin-6 and monocyte chemotactic protein-1 upon stimulation with Hp [1]. This means that pHp, besides of its antioxidative function, plays an immuno-modulatory role in the human lung. This study is intended to further analyze the role of pHp in the human lungs based on these findings.

## Methods

### Materials

This study is in compliance with the Helsinki declaration and was approved by the ethical committee of the University of Lübeck (reference number 03/158); written informed consent was obtained. The collective of patient-samples was the same like in Abdullah et al. 2009 [2]. Here, we used tumor-free lung tissues from patients which underwent surgery due to lung cancer between 2006 and 2008. The patients had a mean age of 65 years (range from 45 to 85 years; 61 males, 48 females). 115 formalin-fixed, paraffin-embedded (FFPE) tissue samples were investigated by immunohistochemistry. The collective contained 47 adenocarcinomas, 42 squamous cell carcinomas, 13 small cell lung cancers and 13 normal lungs. Additionally, we used HOPE-fixed materials from the same collective for immunohistochemistry and confocal microscopy. The numbers of experiments done with each technique are given in the legends of the respective figures.

### Immunohistochemistry

The detection of pHp by immunohistochemistry was performed like previously described [1-3]. FFPE tissues were deparaffinized by a xylene and a graded series of ethanol (2 times 100%, 2 times 96%, 90%, 80%, 70%; 2 times aqua dest.). Hope-fixed tissues were deparaffinized by isopropanol at 60°C and washed with isopropanol at ambient temperature. After air-drying, slides were incubated in 70% acetone (10 min at 4°C), and in water. Endogenous peroxidase was blocked by incubation in 3% H_2_O_2_ for 10 min (HOPE and Formalin). The formalin-fixed tissues were subjected to antigen retrieval (citric buffer pH6, 35 min microwave). Hope-fixed tissues need no antigen retrieval. Anti haptoglobin antibody (clone HG-36, Abcam, Uk) was then applied in a dilution of 1/100 in antibody diluent (Zytomed Systems, Berlin, Germany) for 1 hr at ambient temperature (HOPE and Formalin). Detection was achieved by a polymer system (ZytochemPlus HRP polymer kit, Zytomed Systems, Berlin, Germany) using aminoethlycarbazole as a chromogen. Counterstaining was performed by Mayers hemalum. Negative controls were included in each staining series by omission of the primary antibodies. Sections from human liver served for means of positive control and to ensure even staining throughout the different series.

### Confocal microscopy

The analysis was performed with IL-6-stimulated tissues and A549 cells that have been previously tested for expression of pHp by immunohistochemistry and RNA-in situ hybridization [2]. In short, we used rabbit polyclonal anti-human-Hp (5 μg/ml; Abcam, U.K), and mouse monoclonal anti Surfactant protein-B (SP-B) (clone SPM158 dil. 1:50; Zytomed-Systems, Germany). As secondary antibodies, goat anti-mouse (conjugated with Fluorescein-isothiocyanate, dianova, Germany, 115-095-071) and goat anti-rabbit (conjugated with Texas Red, dianova, Germany, 111-075-144) were applied (both dil. 1:200, 45 min). The mounting medium contained 4',6-diamidino-2-phenylindole. Specimens were analyzed using a Leica TCS SP5 confocal laser scanning microscope (63x plan-apochromatic oil-immersion objective, Leica LA SAF software, Leica Microsystems, Germany). Negative controls were performed by omission of the primary antibodies.

### Immuno-electron-microscopy

Tumor-free (at least 7 cm away from tumor) fresh human lung tissues were obtained as residual material from thoracic surgery due to lung cancer. Small pieces of tissue (approx. 125 mm^3^) were incubated overnight in Roswell Park Memorial Institute medium at 4°C with primary antibodies (Hp: clone HG-36 dil. 1:100, Abcam, U.K.; SP-B: clone SPM158 dil. 1:50, Zytomed-Systems, Germany; Surfactant protein-A clone 32E12, dil. 1:200, Zytomed-Systems, Germany). Negative controls were performed by omission of the primary antibodies. After washing (Phosphate-Buffered Saline, 10 min. twice) tissues were incubated with gold-conjugated secondary antibody (4 nm colloidal gold anti-mouse, dil. 1:100, Jackson, USA, 115-185-068). After washing with Phosphate-Buffered Saline, fixation with glutaraldehyde and embedding in Epon were performed like described previously [4].

## Results

**Figure 1:**
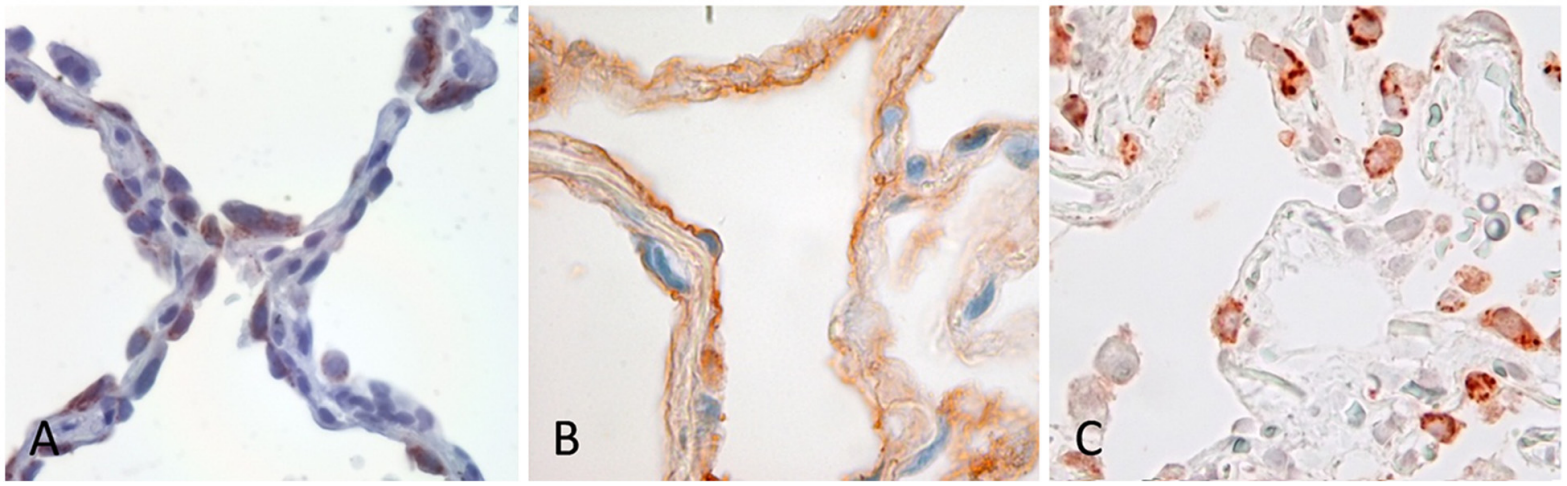
**Immunohistochemistry using different fixation procedures.** Immunohistochemical detection of pHp in human lung tissues using a mouse monoclonal primary antibody and horseradish-peroxidase conjugated polymer for detection with aminoethylcarbazole as a color-substrate. Image-acquisition was performed using a Leica DM-LB2-microscope with 40x PL-Fluotar optics and a Leica DFC 320 camera with Leica software (Leica Microsystems, Germany). White balance, and contrast were adjusted using FixFoto (Koopmann, Germany). **A**: FFPE lung tissue revealing granular staining in AECII (400x). **B**: HOPE-fixed lung tissue revealing ‘greasy’ staining (400x). **C**: HOPE-fixed lung section, which was briefly formalin-fixed after de-paraffinization displaying granular, FFPE-like staining (400x). **A**: one example of 115 FFPE lungs, **B**: one example of more than 15 HOPE-fixed lungs, **C**: one example of more than 15 lungs.

Immunohistochemical detection of pHp in FFPE tissues generally display granular staining-patterns, which implies a deposition of pHp into intracellular vesicles [2]. This granular staining-pattern was observed in AECII (Figure 1A).

While it was not a problem to detect pHp in FFPE-tissues, different results were obtained when HOPE(Hepes, glutamic acid buffer mediated, Organic solvent Protection Effect)-fixed, paraffin embedded tissues were subjected to pHp immuno-stainings [2]. These generally showed ‘greasy’ staining patterns which, in the first view, looked like ‘background’ (Figure 1B). Curiously, even extended optimization of the staining protocols did not change these results. The preservation of protein structures in HOPE-fixed materials has been repeatedly shown to be clearly superior to FFPE, which - besides application of ‘cryo-type’ antibodies in immunohistochemistry - enables sophisticated techniques like the detection of signal-cascade-molecules in Western blots or even proteomic approaches to be applied to such materials [3,5-7]. This was a crucial prerequisite for the development of a human ex vivo model for the short term stimulation of lung tissues [8-10]. Keeping in mind the biologic functionality of HOPE-fixed materials, we hypothesized that pHp might be secreted from lung cells and could still be released from the de-paraffinized sections. The analysis of secretion of pHp using human lung explants and A549 cells showed a fast secretion of pHp in unambiguously detectable amounts in both systems [1]. As a next step de-paraffinized HOPE-sections were shortly subjected to formalin (2 min. in 4% neutral buffered formalin), and then stained for pHp. Here, the ‘background’ disappeared and clear FFPE-like pHp-immuno-stainings were observed (Figure 1C).

**Figure 2:**
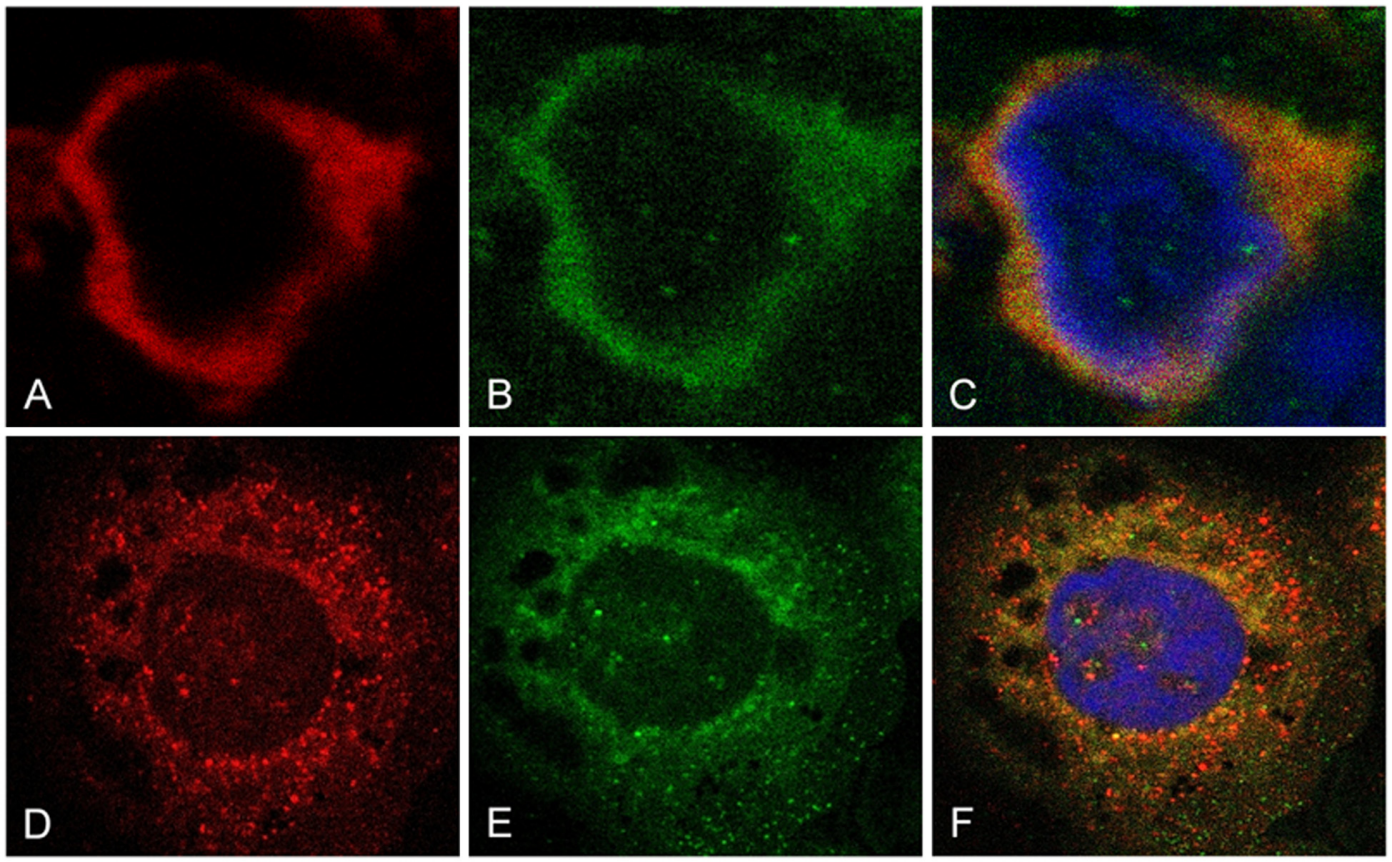
**Confocal microscopy.** Double immuno-staining for pHp and SP-B using a confocal microscope (Red: pHp, Green: SP-B, Blue: Nuclei stained with 4',6-diamidino-2-phenylindole). **A**-**C**: AEC II in a HOPE-fixed human lung tissue. **A**: pHp; **B**: SP-B; **C**: overlay of all three channels displaying co-localization of pHp and SP-B in orange color (1000x). **D**-**F**: A549 cells treated in the same way as **A**-**C** (1000x). Two independent experiments.

We performed confocal microscopy to address the question of a possible co-localization of pHp with surfactant proteins. We found a complete co-localization of pHp with SP-B in AECII in lung tissues and a high, but not complete, portion of co-localization in A459 cells (Figure 2A-C and D-F).

**Figure 3:**
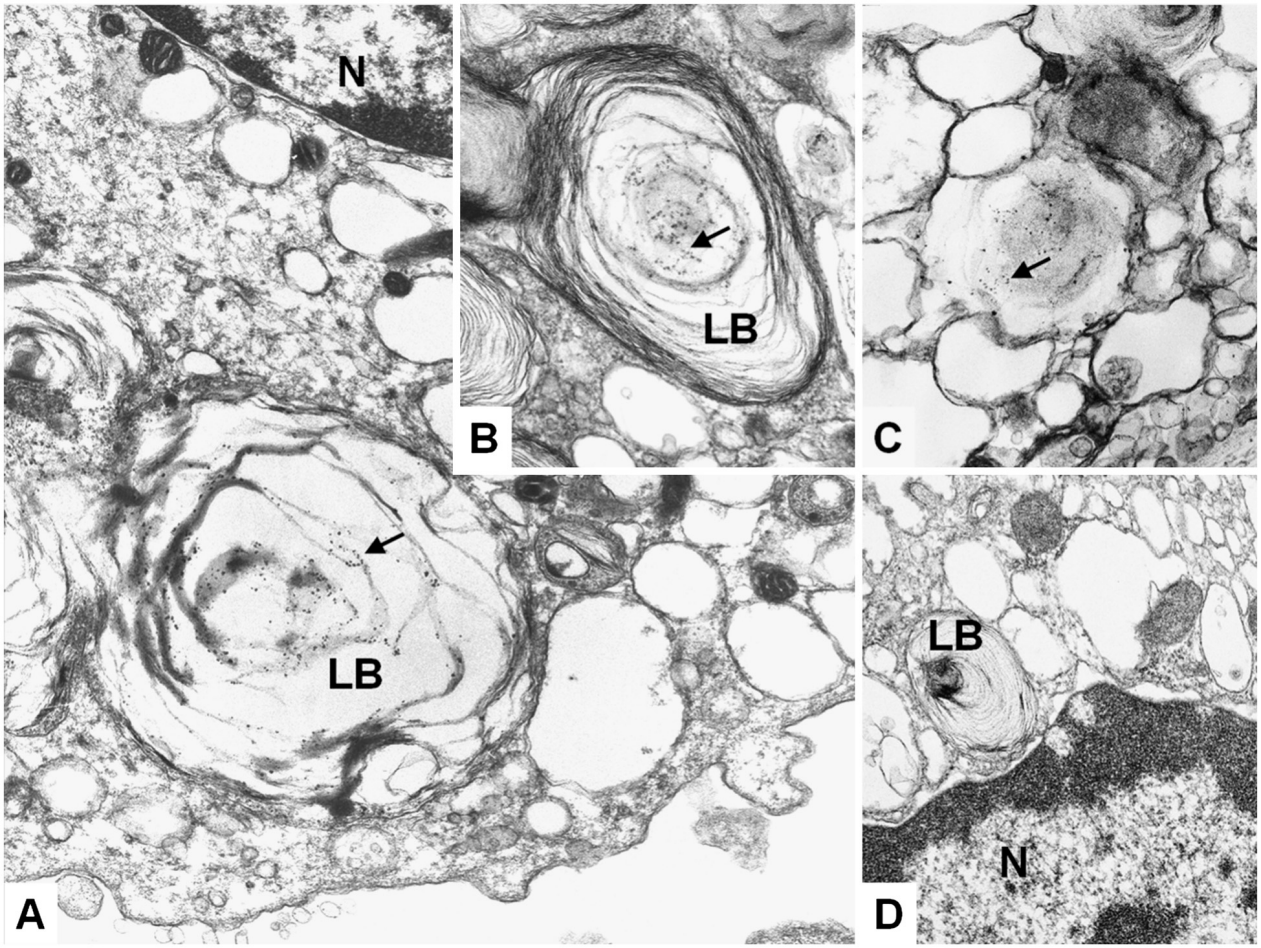
**Immuno-electron-microscopy.** Immuno-electron-microscopic detection of pHp, SP-B and SP-A (LB: lamellar body, N: nucleus). Image-acquisition was performed using a Zeiss EM 910 transmission-electron-microscope with built in analogue camera (Carl Zeiss, Germany). Negatives were scanned with a Flextight X5 scanner with FlexColor software version 4.8.8. (Hasselblad, Sweden). **A**: pHp in a lamellar body (5000x). **B**: SP-B in a lamellar body (5000x). **C**: Surfactant protein-A in a not lamellar vesicle (5000x). **D**: Negative control (5000x). Two independent experiments.

To confirm this data, immuno-electron-microscopy was performed and pHp was detected within lamellar bodies of AECII (Figure 3A). SP-B, as expected, was also found to be located in lamellar bodies of AECII applying the same technique as used for pHp (Figure 3B). Surfactant protein-A, also as expected, was found in vesicles which are not lamellar bodies (Figure 3C), while negative controls under omission of primary antibody remained negative (Figure 3D). Therefore, pHp and SP-B are co-localized within the lamellar bodies of AECII, which are known to be a source of surfactant.

**Figure 4:**
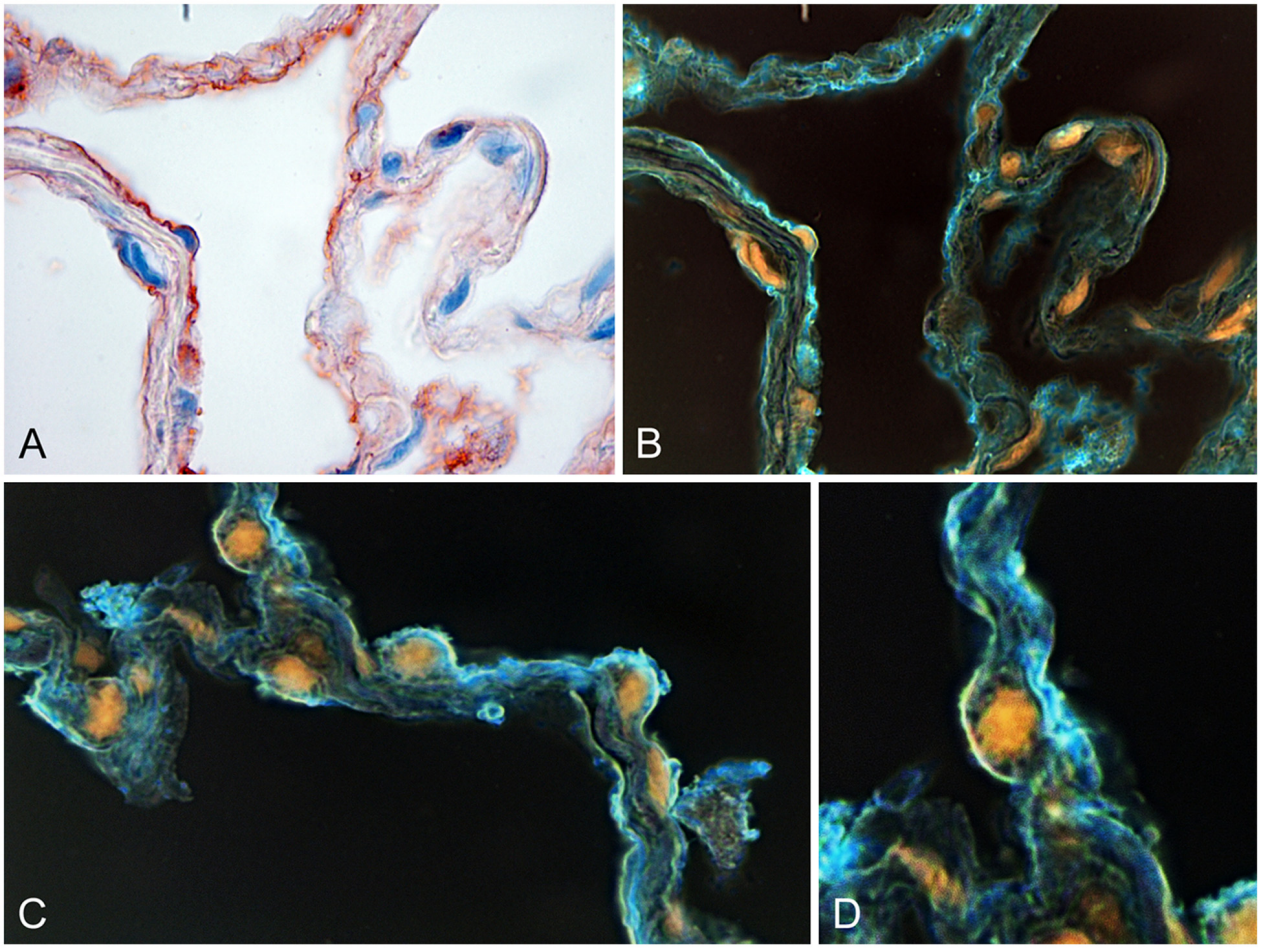
**False-color imaging.** Immunohistochemical detection of pHp in HOPE-fixed, paraffin embedded human lung tissues. Image-acquisition was performed using a Leica DM-LB2-microscope with 40x PL-Fluotar optics and a Leica DFC 320 camera with Leica software (Leica Microsystems, Germany). White balance, and contrast were adjusted using FixFoto (Koopmann, Germany). **A**: Same as Figure 1B (400x), **B**: the same specimen with color inversion; pHp now in blue color (400X), **C** and **D**: Two other examples of color-inverted photomicrographs showing a film of pHp in the intended position of the surfactant in human alveoli (400x). Examples of more than 10 experiments.

We finally re-analyzed the ‘greasily’ stained HOPE-fixed sections and found, by application of a simple and color-inversion (FixFoto, Koopmann, Germany), a fine blue film of pHp-stained material lining the alveolar surface (Figure 4). Amazingly, this film exactly resembles the intended distribution of pulmonary surfactant.

## Discussion

Recently, haptoglobin has been described in the lung in studies which applied proteomic technologies, suggesting that the awareness of its biological function in the lung is increasing [11-16]. If we look back to the basics of knowledge about surfactant, its preparation and the proteins inside these preparations, Kevin M.W. Keough summarizes this issue in the first chapter of the book “Lung Surfactant: Cellular and Molecular Processing” in the following way: “Surfactant is usually prepared in the laboratory from material lavaged from the lung by different and density gradient centrifugations that are intended to remove extraneous material such as plasma proteins, which are considered unlikely constituents of normal surfactant, since they inhibit its actions. Depending upon the extent of “purification” of the material, however, it is possible, if unlikely, that some materials which may in effect be part of surfactant “as the cell sees it” might be lost in the process” [17]. Hp, however, is a plasma protein, and therefore will have been removed from the surfactant preparations, which is why it remained undiscovered as a possible part of this system.

Taken together, we provided proof that pHp is a surfactant protein, which is a new function of this long known molecule. Due to the antioxidant activity the presence of pHp within the surfactant film can be involved in fast repair of arterial leakages. The direct surface-activity of pHp remains to be analyzed. Since pHp removes free hemoglobin which has surface activity [11], there is at least an indirect influence of pHp on the surface activity in the human lung. The immuno-modulatory facilities of pHp complement the activity of the other four surfactant proteins. Furthermore, inflammatory and infectious events in the human lung take place in a pHp-enriched environment. We are confident that this will add to a better understanding of the complex immuno-regulatory events in the lung and can contribute to develop novel therapeutic strategies for different lung diseases in the future.

## Competing interests

The authors declare that they have no competing interests.

## Authors’ contributions

MA did the experiments and was involved in finalizing the manuscript. TG conceived of the study and wrote the manuscript. All authors read and approved the final manuscript.
